# Pamphlets Received

**Published:** 1855-03

**Authors:** 


### *Pamphlets Received.*

*Annual Report of the Commissioners and Superintendent of the Indiana Hospital for the Insane.*

A comprehensive and interesting document.

*Transactions of the Illinois State Medical Society, for the year* 1854.

A good sized pamphlet, and indicative of a high state of cultivation in the energetic West. Dr. Brainard's address on the subject of poisoned wounds, begins the volume, and is followed by an interesting paper on "Walking rendered the Primary Element in the Cure of Deformities of the Lower Extremities; its early adaptation to White Swelling and Coxalgia, with apparatus for carrying out the designs of the same." By E. S. Cooper, M. D., Peoria, Ill. The Prize Essay is by H. Parker, M. D., of Chicago, on "The

Differences in the Pathological and Physiological Action of Stimulants and Tonics," a very well reasoned effort.

*Table of Urinary Deposits, with their Microscopical and Chemical Tests, for Clinical Observations.* By John King, M. D., Cincinnati, Ohio.

This "cometh from Nazareth," but its design is good, its chemistry correct, and it is altogether a convenient thing for daily reference in qualitative analysis of urine.

*Essay on a New Method of Treating Serpent Bite and other Poisoned Wounds. Being the Annual Address delivered before the Illinois State Medical Society, June 7th,* 1854. By Daniel Brainard, M. D.

Dr. Brainard has made many careful experiments upon poisoned wounds. He corrects the general opinion that serpent bites are usually fatal. The larger proportion of cases recover without treatment. He denies the "cure by intoxication," of which so much has been said, partly on the ground of an insufficiency of evidence, and partly because, "1st. When mixed with alcohol the venom is rapidly fatal. 2d. Alcohol injected into the tissues, or introduced into the stomach of birds, or of small animals which have been bitten, hastens death. This circumstance is not conclusive, as alcohol is of itself a poison to them. 3. Persons bitten by rattle-snakes when in a state of intoxication are not, on that account, secure. I have authentic information of four cases in which the bite of that snake proved rapidly fatal on intoxicated persons. Another may be found related in the Amer. Jour. of Med. Sciences, vol. 8, 1831."

Dr. Brainard has great confidence in the injection of iodine into the tissues. As the fang of the serpent discharges its poison at the bottom of a wound half an inch in depth, a mere external application is not sufficient. The tube of a small syringe should be passed to the bottom of the wound, and the tincture thoroughly infiltrated in the tissues.

*Sanitary Reports of the City of Buffalo for the year* 1854. *Printed by order of the Common Council.*

A very neat pamphlet of some sixty pages, embodies the Health Physician's Cholera Report, the statistics of the Small-Pox Hospital, and the accounts of expenditure for the year.

Dr. Newman, the Health Physician for the last year, entered upon his office with zeal and an intelligent understanding of the means by which it might be made useful to the profession. Aside from the strictly scientific researches into the phenomena of cholera, and the protective influence of vaccination, in which he engaged, he gave vigor and life to the sanitary police of our city. The whole conduct of the Board of Health for 1854, was marked by energy, zeal, and a wise foresight.

The report informs us that as early as April there were evidences of the choleraic influence, but no fatal case occurred till May 23d. Deaths occurred also on the 5th, the 15th, and 21st of June. From this latter date cases occurred daily: the number of cases in June being 17.

After the 1st of July cases occurred in every part of the city, in such widely separated localities, as to indicate its epidemic character.

A number of very interesting tables are given on the connection of humidity with cholera. These tables are so arranged as to show at a glance, at any given day, the height of the barometer, the quantity of rain, amount of clouds, the force and direction of winds, the temperature, dew-point, and humidity, at 2, P. M., the number of cases of cholera, the number of deaths, and the number of cases sickening on that day which subsequently proved fatal; and finally, the number of cases and deaths at the Almshouse.

It will be noticed that these tables are very complete, and though the deductions from them seem somewhat contradictory, they are still valuable.

After running a parallel between cholera and the dew-point, during the early days of July, the report calls attention to "the increase of temperature to 80° on the 10th, and remaining above that point till the evening of the 25th; and the sudden increase of the dew-point, on the 19th, to 72.33°, with a corresponding development of the disease. From this period to the end of the month there was an increase of it in the city. During the same interval of time it raged with fearful violence at the Almshouse; and broke out at the Suspension Bridge, Niagara Falls, a perfect pestilence."

Evidence is not wanting to show such a connection as Dr. Newman indicates, but it is unnecessary to say to those who have carefully investigated this matter, that the relations of cholera to temperature are very wide and uncertain, while its relations to humidity are only less so. Cholera occurred late in November in this city, in the family of a respectable physician living in all the comforts of life, when the thermometer marked only 35°. So far as temperature is concerned it may occur from the climate of winter to the heats of summer.

Of course with such a thermal variation we have a corresponding difference in aqueous vapor. It should, however, be borne in mind, that these are exceptions to the rule, and that cholera is a disease of hot and humid climates. It cannot be contended that humidity stands in the relation of a direct cause, but it is certainly one of the exciting causes, a cause of the last importance, giving efficacy and virulence to all other exciting causes, and materially influencing the progress of all zymotic disease.

Second to these general causes, Dr. Newman inquires into the special causes which determined the ravages of the epidemic to certain localities. Mortimer st., the Hydraulics, South Division st., and the Poorhouse, come in for a rigid examination. It is the old story with few exceptions. Diet, deprivation, intemperance, claim their victims in every pestilence. The report does not spare them, and of all the so-called "libellous" articles on the Poorhouse mortality, this report is the most caustic.

From a table giving the streets, with the No. of cases on each, we gather that there occurred at

| | | |
|---|---|---|
| The Almshouse, - - - - | cases, | 104 |
| Streets not given, - - - - - | " | 42 |
| Emigrants and Travelers, - - - | " | 74 |
| On paved streets, - - - - - | " | 327 |
| On unpaved streets, - - - | " | 408 |
| Total, - - - | | 1035 |

It will be seen that of 1035 cases, 327 occurred on paved streets, in a city which is mostly paved. Of these 327, Genessee street, (German and not cleanly,) is credited with 61 cases; South Division with 28; N. Division 13; Seneca 32; and Exchange 21; making 154 in localities where the causes were evidently chargeable to local surroundings.

Of the small-pox statistics it is only necessary to give the result. During the year, 58 cases were admitted to the hospital, of whom 2 died.

The total number of deaths from all causes was 2936. Estimating the population at 80,000, the proportion of deaths to inhabitants was as 1 in 27.21. This is a heavy average, though not so bad for a cholera year. The healthiest city in the world, London, has a ratio of 1 in 40. Few American cities have a ratio of less than 1 in 30. The causes of this large mortality may be reached.

There is a large foreign population in Buffalo. We will estimate the native population at 50,000, and assign the deaths of each class to their numbers.

Of 2936 deaths the country is given as follows:

| | |
|---|---|
| Americans, - - - - - - | 889 |
| Foreigners, - - - - - - - | 1483 |
| Country not given, - - - - | 574 |

If we assume that the cases in which the country is not given may be equally proportioned to the two classes, then of this 574 deaths, 346 should be added to the American deaths, and 228 to the foreign deaths, leaving the number of Americans 1117, and of foreigners 1819.

Dividing 50,000 by 1117 will give us the ratio of American deaths to population, as 1 in 44.76; and dividing 30,000 by 1819, will give us the ratio of deaths to foreign population as 1 in 16.49.

Of course there are the usual liabilities to error in this calculation. The proportions of the population are estimated — not known — but even were we to charge the whole unknown nationality of 574 deaths to the American loss, we should have only the following ratio: 50,000÷1463=34.11, an average such as few cities can boast.

Appended to the report is a table giving in the order of precedence of time, the streets on which cholera occurred, and the number of cases on each day, thus showing the order in which localities were attacked, and the length of time elapsing before the disappearance of the disease from them.

The report is a credit to its able author, Dr. Newman, and to the city authorities who gave the means for the acquisition of facts and their publication. H.

